# Percutaneous closure of patent foramen ovale after cryptogenic stroke – a comparison between patients < 60 versus ≥ 60 years-of-age

**DOI:** 10.1007/s12928-026-01269-z

**Published:** 2026-04-29

**Authors:** Anna Damlin, Dinos Verouhis, Magnus Settergren

**Affiliations:** 1 Department of Cardiology, Karolinska University Hospital, Karolinska University Hospital, Stockholm, Sweden; 2 Department of Molecular Medicine and Surgery, Karolinska Institutet, Stockholm, Sweden; 3 Department of Medicine Solna, Karolinska Institutet, Stockholm, Sweden

**Keywords:** Patent foramen ovale, Cardiovascular interventions, Ischemic stroke

## Abstract

Cryptogenic ischemic stroke is strongly associated with patent foramen ovale (PFO). Current guidelines recommend percutaneous PFO closure in patients < 60 years-of-age. This study aimed to compare the incidences of recurrent stroke or transient ischemic attack (TIA), and atrial fibrillation after percutaneous PFO closure in patients aged *≥* 60 years with patients < 60 years-of-age. This retrospective study included all adult Swedish patients with cryptogenic stroke treated with percutaneous closure of PFO 2001–2023. Patient characteristics and procedural details were obtained from the Swedish registry of adult congenital heart disease. Preprocedural atrial fibrillation and post-procedural incidence of recurrent cerebral infarction, TIA, and atrial fibrillation were obtained from the Swedish National Board of Health and Welfare’s registry of patient diagnoses. To compare differences across age groups, logistic-, quantile-, and linear regression models were used. P-values < 0.050 were considered significant. In total, 1,101 patients were included with follow-up time 7.1 ± 5.8 years, of which 12% (*n* = 134) were *≥* 60 years-of-age. Low prevalence of preprocedural comorbidity was seen in both age groups and there was no difference in incidence of atrial fibrillation within 3 months after PFO closure (*≥* 60 years-of-age *n* = 4, 3.0%, < 60 years-of-age *n* = 23, 2.4%, *p* = 0.229), or recurrent cerebral infarction/TIA (*≥* 60 years-of-age *n* = 3, 2.7%, < 60 years-of-age *n* = 17, 1.5%, *p* = 0.697) between the age groups. Based on the results from this nation-wide study, percutaneous PFO closure can be considered a safe and efficient therapy in the reduction of recurrent stroke in patients older than 60 years with low preprocedural comorbidity.

**Graphical Abstract:**

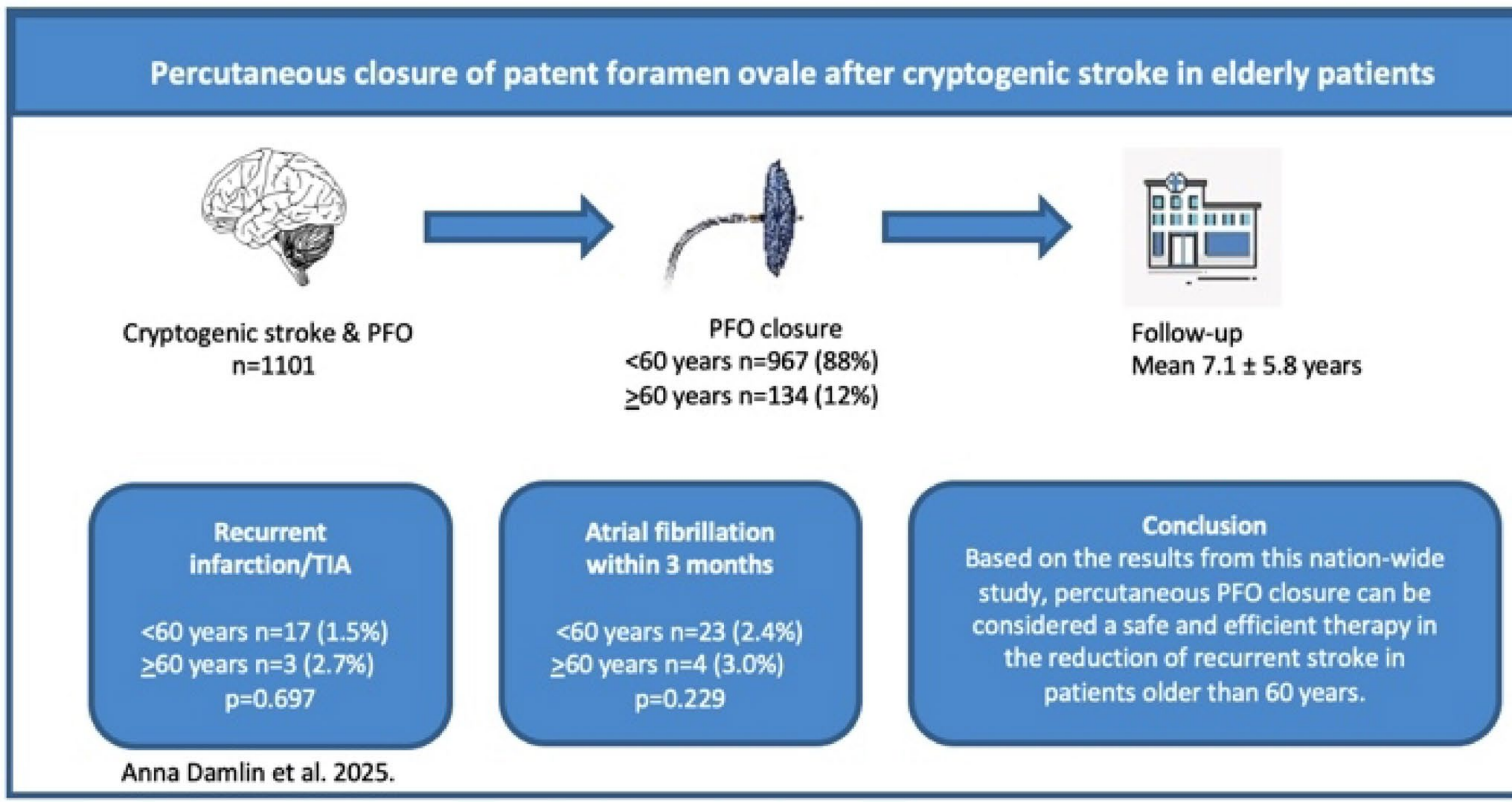

**Supplementary Information:**

The online version contains supplementary material available at 10.1007/s12928-026-01269-z.

## Background

Up to a third of all ischemic strokes are categorized as cryptogenic, i.e., unexplained infarcts (1, 2). There is a strong association between cryptogenic stroke and the presence of patent foramen ovale (PFO), suggesting paradoxical embolism as an important underlying mechanism for ischemic stroke in these patients (3). PFO is more common in cryptogenic stroke both in patients < 55 years (44–50%), and in elderly patients (28%), compared to patients with stroke due to other causes (14% in patients < 55 years and 12% in patients > 55 years) (4). Randomized clinical trials demonstrate benefits with percutaneous PFO device closure, compared with medical therapy in patients *≤* 60 years-of-age (2, 5–8) and guidelines state that patients with cryptogenic stroke and PFO younger than 60 years should be suggested for percutaneous PFO closure (9). It has been shown that elderly patients with cryptogenic stroke and PFO have significantly lower prevalence of atherosclerotic risk factors, atrial fibrillation, and risk of systemic embolic events, compared with patients with other types of stroke (10). Factors contributing to an elevated risk of paradoxical emboli in the elderly are increased risk of deep vein thrombosis due to changes in the endothelial and platelet function, comorbidities, reduced physical activity; and increased diameter of the PFO due to greater filling pressure of the ventricles favoring a right-to-left shunt (11). Further, elderly patients may even be at higher risk of PFO-related recurrent stroke compared with younger patients (12, 13). With an increasing number of interventions performed during the last decades, the success rates of PFO closure have increased, and may therefore render less recurrent strokes due toresidual shunts (5–9). Studies analyzing PFO closures in patients aged > 60 years have shown similar rates of procedural success and no significant differences in recurrent stroke or transient ischemic attack (TIA) compared to patients younger than 60 years, although elderly patients have shown higher risk developing post-procedural atrial fibrillation (14). Especially elderly with cryptogenic stroke and a high-risk PFO (e.g., atrial septal aneurysm and/or a large-sized shunt) may significantly benefit from undergoing percutaneous PFO device closure to prevent recurrent ischemic events (13). A study of effectiveness derived from major observational studies presents an anticipated relative reduction in recurrent stroke of approximately 50% in older patients with cryptogenic stroke and high-risk PFO (15). However, the follow-up periods for the studies of patients > 60 years undergoing PFO closure are limited (Ben-Assa et al.: 3.6 years, Hyung Lee et al.: 3.9 years), and the European Stroke Organization has recently stated that there is yet insufficient evidence to make an evidence-based recommendation on PFO closure in patients > 60 years (16). Hence, there is a need for an updated evaluation of patients aged *≥* 60 years, with cryptogenic stroke and PFO, to evaluate the long-term safety and efficacy of PFO closure in this patient group.

## Aim

To evaluate the short- and long-term safety and efficacy of percutaneous PFO closure in patients aged *≥* 60 years with cryptogenic stroke, by comparing the incidences of recurrent stroke or TIA, atrial fibrillation, and paradoxical embolization with those in patients < 60 years-of-age.

## Methods

### Study population

This retrospective, registry-based study aimed to include all adult Swedish patients treated with percutaneous PFO closure between December 1^st,^ 2001, and April 30th, 2023, after cryptogenic stroke. The study population was obtained from the Swedish registry of adult congenital heart disease (SWEDCON), selecting patients that were registered with percutaneous PFO closure in Sweden. Exclusion criteria were congenital heart disease or interrupted intervention, no registered diagnosis of cryptogenic stroke, and incomplete PFO closure device information.

### Data collection

Patient characteristics, procedural data, and follow-up data were collected from SWEDCON. Comorbidity (i.e. preprocedural prevalence of cancer, chronic obstructive pulmonary disease, deep venous thrombosis, diabetes mellitus, heart failure with reduced ejection fraction, ischemic heart disease, hyperlipidemia, hypertension, pulmonary emboli, and renal insufficiency) were collected as registry-based data, i.e. if these diagnoses were listed as diagnosis codes in the SWEDCON registry. Preexisting atrial fibrillation and post-procedural incidence of recurrent cerebral infarction, TIA, peripheral embolization, and atrial fibrillation were obtained from the Swedish National Board of Health and Welfare’s registry of patient diagnoses, using the ICD codes listed in Table S1. The diagnoses of stroke and TIA were verified by the centers of procedure. Follow-up time was calculated as time from the PFO closure to the last day of the study period or to the date of death if that occurred during the study period.

### Statistical analysis

Patient characteristics (age, sex, comorbidity, previous cardiac interventions/surgery, peri- and post-operative complications, date, and details from the follow-up, and mortality) were described on a group level. Procedure related details (date of intervention, device type and size, procedural duration, fluoroscopy time, type of echocardiographic imaging, type of anesthesia) from the percutaneous PFO closures and long-term outcomes were described and compared between the different age groups (*≥* 60 years-of-age versus < 60 years-of-age). To verify if continuous data were normally distributed, the Shapiro-Wilk test was used. Normally distributed data are presented with mean ± standard deviation (SD). Non-normally distributed data are presented with median and interquartile range (IQR). Binary variables are reported as absolute numbers and percentages. To compare differences across groups, logistic- (binary variables), quantile- (non-normally distributed continuous variables), and linear regression (normally distributed continuous variables) models were used. P-values < 0.050 were considered significant. Data analysis was conducted using STATA software (version 17.0 Stata Corp., College Station, Texas, USA).

## Results

**Fig. 1:**
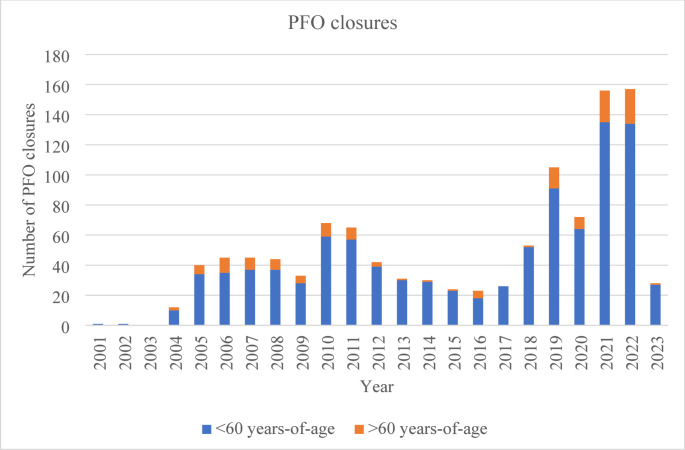
Number of patients with cryptogenic stroke undergoing percutaneous PFO closure. presents number of PFO closure procedures each yearduring the study period in total and in each age group

In total, 1,101 patients (38% women) with cryptogenic stroke underwent percutaneous PFO closure in Sweden during the study period, of which 134 (12%) were *≥* 60 years-of-age (Table 1), Fig 1). The follow-up period was 7.1 ± 5.8 years (median 4.9, IQR 1.9–12.3). The prevalence of comorbidities was generally low in the study population and did not differ between the age groups (Table 1). However, the prevalence of deep venous thrombosis was higher in the *≥* 60 years group (3% versus 1%, *p* = 0.023) (Table 1). Smoking, or history of previous smoking was more common in the *≥* 60 years group (28.7% versus 19.8%, *p* = 0.041). There was no difference in prevalence of pre-procedural left-to-right directed shunts between the age groups (Table S2).

**Table 1:** Patient characteristics and comparisons between the different age groups of patients with cryptogenic stroke undergoing percutaneous PFO closure

	All patients, *n* = 1,101	*≥* 60 years-of-age, *n* = 134	< 60 years-of-age, *n* = 967	*p*-value
Age, median (IQR)	48.4 (40.2–55.4)	63.3 (61.3–65.4)	46.8 (38.7–52.7)	
Women, n (%) Men, n (%)	419 (38) 682 (62)	44 (33) 90 (67)	375 (39) 592 (61)	0.185 0.185
**Comorbidities prior to PFO closure, n (%)**				
Cancer COPD Deep venous thrombosis Diabetes Atrial fibrillation HFrEF Ischemic heart disease Hyperlipidemia Hypertension Pulmonary emboli Renal insufficiency	3 (0) 1 (0) 11 (1) 5 (0) 11 (1) 1 (0) 8 (1) 12 (1) 46 (4) 15 (1) 2 (0)	1 (1) 1 (1) 4 (3) 0 (0) 2 (2) 0 (0) 1 (1) 3 (2) 13 (10) 3 (2) 0 (0)	2 (0) 0 (0) 7 (1) 5(1) 9 (1) 1 (0) 7 (1) 9 (1) 33 (3) 12 (1) 2 (0)	0.294 - **0.023** - 0.395 - 0.977 0.186 0.853 0.519 -

The p-values are generated from the comparisons of the different age groups, i.e. quantile regression comparing continuous variables and logistic regression comparing binary variables. Abbreviations: COPD: chronic obstructive pulmonary disease, CVC: cardiovascular comorbidity, HFrEF: heart failure with reduced ejection fraction, IQR: interquartile range, PFO: patent foramen ovale

The most common device used for PFO closure was the Amplatzer device (*n* = 620, 56%), and this device was more commonly used in the *≥* 60 years-of-age group (*n* = 89, 66%) compared with the < 60 years-of-age group (*n* = 531, 55%, *p* = 0.012) (Table 2). The GORE^®^ Cardioform Septal Occluder device was more commonly used in the < 60 years-of-age group (*n* = 313, 32%) compared with the *≥* 60 years-of-age (*n* = 28, 21%, *p* = 0.008). The distribution of device sizes is presented in Table S3. The use of devices > 25 mm was more common in the *≥* 60 years-of-age group (*n* = 51, 38%) compared with the < 60 years-of-age group (*n* = 279, 29%, *p* = 0.030). Among the devices > 25 mm, 62.4% were Amplatzer devices, and 36.3% were GORE^®^ Cardioform Septal Occluder.

**Table 2:** Periprocedural details of percutaneous PFO closures in patients with cryptogenic stroke

	All patients, *n* = 1,101	*≥* 60 years-of-age, *n* = 134	< 60 years-of-age, *n* = 967	*p*-value
Procedural time, median minutes (IQR)	55.0 (40.0–70.0)	60.0 (45.0–72.5.0.5)	55.0 (40.0–70.0)	0.030
Fluoroscopy time, median minutes (IQR)	9.0 (6.0–13.0)	11.0 (7.0–15.0)	9.0 (6.0–13.0)	**< 0.001**
General anesthesia, n (%)	417 (38)	52 (39)	356 (38)	0.813
Guided by TTE/TEE/ICE, n (%)	1,084 (98)	133 (99)	951 (98)	0.475
**Devices for PFO closure**				
Amplatzer PFO occluder, n (%) GORE^®^ Cardioform Septal Occluder, n (%) Helex, n (%) Occlutech Figulla, n (%) NobleStitch, n (%) Solysafe, n (%)	620 (56) 341 (31) 90 (8) 28 (3) 19 (2) 3 (0)	89 (66) 28 (21) 11 (8) 3 (2) 3 (2) 0 (0)	531 (55) 313 (32) 79 (8) 25 (3) 16 (2) 3 (0)	**0.012** **0.008** 0.988 0.811 0.628 -

The p-values are generated from the comparisons of the different age groups, i.e., quantile (including median) regression comparing continuous variables and logistic regression comparing binary variables. Abbreviations: ICE: intracardiac echocardiography, IQR: interquartile range, PFO: patent foramen ovale, TOE: transesophageal echocardiography, TTE: transthoracic echocardiography

**Fig. 2:**
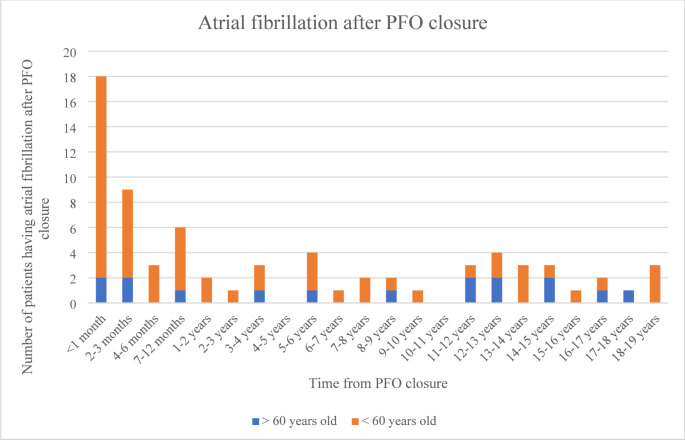
Post-procedural atrial fibrillation in patients with cryptogenic stroke undergoing percutaneous PFO closure. presents time from PFO closure tofirst onset of post-procedural atrial fibrillation

Both the procedure duration and fluoroscopy time were longer in the *≥* 60 years-of-age group (Table 2). The post-procedural success rate of PFO closure was 96.5%. One patient (< 60 years-of-age) had an embolization of the closure device 7 days post-procedure and was surgically treated with extirpation of the embolized device and surgical closure of the PFO with a patch. In total, 3 patients were registered with a redo PFO closure due to residual shunt and recurrent ischemic stroke. This occurred with an average of 2.0 years post procedure. In total 71 (6%) patients had postprocedural atrial fibrillation (Table 3; Fig. 2), with a significantly later onset in the *≥* 60 years-of-age group. Postprocedural atrial fibrillation was more common in the *≥* 60 years-of-age group (Table 3). Of the patients with postprocedural atrial fibrillation, 27 (2.5%) were registered within 3 months after the procedure, 4 (3.0%) in the *≥* 60 years-of-age group and 23 (2.4%) in the < 60 years-of-age group, with equal distribution in the age groups (*p* = 0.229). A timeline of the onset of post-procedural atrial fibrillation is shown in Fig. 2. The incidence of new-onset atrial fibrillation > 3 months after closure, was 9.5 per 1,000 person-years in the *≥* 60 years-of-age group and 4.6 per 1,000 person-years in the < 60 years-of-age group. Post-procedural atrial fibrillation was more common in patients who had a device size *≥* 20 mm, compared with < 20 mm (*p* = 0.037).

**Table 3:** Events during follow-up in patients with cryptogenic stroke undergoing percutaneous PFO closure

	All patients, *n* = 1,101	*≥* 60 years-of-age, *n* = 134	< 60 years-of-age, *n* = 967	*p*-value
**Postprocedural events, n (%)**				
Follow up time, median years (IQR)	4.9 (1.9–12.3)	4.1 (1.9–12.6)	4.9 (1.9–12.2)	0.512
Atrial fibrillation, n (%) Atrial fibrillation *≤* 3 months after closure, n (%) Days from closure to atrial fibrillation, median (IQR)	71 (6.4) 27 (2.5) 353.0 (25.0–4149.0.0.0)	16 (11.9) 4 (3.0) 3643.5 (180.0–4972.0.0.0)	55 (5.7) 23 (2.4) 229 (21.0–2841.0.0.0)	**0.013** 0.229 **0.002**
Cerebral infarction/TIA Peripheral embolism ICB Embolization of device Bleeding Tamponade Infection	20 (1.8) 2 (0) 0 (0) 1 (0) 0 (0) 0 (0) 1 (0)	3 (2.7) 0 (0) 0 (0) 0 (0) 0 (0) 0 (0) 0 (0)	17 (1.5) 2 (0) 0 (0) 1 (0) 0 (0) 0 (0) 1 (0)	0.697 - **-** **-** **-** **-** **-**
**Reintervention**,** n (%)**	3 (0)	0 (0)	3 (0)	-

Periprocedural complications refer to complications that occurred during the percutaneous procedure. Postprocedural complications refer to complications that occurred after the procedure. Non-normally distributed data are presented with median and interquartile range (IQR). The p-values are generated from the comparisons of the different age groups, i.e. quantile (including median) regression comparing continuous variables and logistic regression comparing binary variables. Statistically significant p-values are marked in bold. Abbreviations: AF: atrial fibrillation, ICB: intracranial bleeding, TIA: transient ischemic attack

After PFO-closure, 20 patients (1.8%) were registered with either cerebral infarction (*n* = 16, 1.5%) or TIA (*n* = 4, 0.4%), 3 (0.3%) within a year after the PFO closure. All patients with recurrent cerebral infarction or TIA within a year after the PFO closure were < 60 years of age. Four of the 20 patients registered with either cerebral infarction or TIA had atrial fibrillation registered prior to, or at the time when the infarction or TIA was revealed. The incidence of recurrent stroke was 2.8 per 1,000 person-years in the *≥* 60 years-of-age group and 2.5 per 1,000 person-years in the < 60 years-of-age group. Additionally, two patients were registered with peripheral emboli after PFO closure. Both occurred approximately 4 years after the procedure.

Anticoagulative treatment was more common among patients *≥* 60 years of age compared with the patients < 60 years of age, both regarding pre-procedural treatment (*≥* 60 years of age: *n* = 40, 30%, < 60 years-of-age: *n* = 214, 22%, *p* = 0.017), and post-procedural treatment (*≥* 60 years of age: *n* = 31, 23%, < 60 years-of-age: *n* = 147, 15%, *p* = 0.009). Antithrombotic treatment was equally common in the two age groups, both regarding pre-, and post-procedural treatment (Table 4).

**Table 4:** Treatment details of percutaneous PFO closures in patients with cryptogenic stroke

	All patients, *n* = 1,089	*≥* 60 years-of-age, *n* = 132	< 60 years-of-age, *n* = 957	*p*-value
**Preprocedural treatment**				
Antithrombotics	668 (61)	72 (55)	596 (62)	0.105
ASA	545 (50)	57 (43)	488 (51)	
Other antithrombotics	123 (11)	15 (11)	108 (11)	
Anticoagulants	254 (23)	40 (30)	214 (22)	**0.017**
DOAC	6 (1)	1 (1)	5 (1)	
Warfarin	248 (23)	39 (30)	209 (22)	
**Postprocedural treatment**				
Antithrombotics	746 (69)	84 (64)	662 (69)	0.836
ASA	595 (55)	61 (46)	534 (56)	
Other antithrombotics	151 (14)	23 (17)	128 (13)	
Anticoagulants	178 (16)	31 (23)	147 (15)	**0.009**
DOAC	2 (0)	1 (1)	1 (0)	
Warfarin	176 (16)	30 (23)	146 (15)	

Values presented in number and percentage in parenthesis. The p-values are generated from the comparisons of the different age groups, using logistic regression. Eleven patients did not have medication registered in the registry. Abbreviations: ASA: acetylsalicylic acid, DOAC: direct oral anticoagulants, PFO: patent foramen ovale

During the follow-up, 27 (2%) patients died (mean 9.0 ± 4.9 years after PFO closure), of which 1 patient died within a year after the PFO closure (85 days post-procedure of suspected ventricular arrhythmia, in the < 60-years-of-age group). Of the 27 patients, 6 were registered with cardiac death but none underwent autopsy. The rate of cardiac death was similar in the two age groups (*n* = 2 in the *≥* 60 years-of-age group and *n* = 4 in the < 60-years-of-age group, *p* = 0.313). The mean age of the patients that died was 68 ± 9 years, and 14 (52%) of them were *≥* 60 years-of-age at the time of the PFO closure.

## Discussion

Percutaneous PFO closure is established for prevention of recurrent cerebral ischemic events in patients with a history of cryptogenic stroke and PFO, primarily based on three randomized trials (5, 7, 8). However, these existing trials are based on study populations under the age of 60 years (2, 5–8), and consequently guidelines specifically recommend percutaneous PFO closure in this age group (9). There is a need for studies on large populations with long follow-up, to evaluate the possible benefits for PFO closure in patients older than 60 years. This present nation-wide study covers all percutaneous PFO closures (*n* = 1,101, 134 patients *≥* 60 years) after cryptogenic stroke performed in adult patients over a 22.4-year study period with mean follow-up 7.1 years (median 4.9 years). It shows that patients *≥* 60 years-of-age with a history of cryptogenic stroke and PFO, that underwent percutaneous PFO closure had no elevated risk of early-onset atrial fibrillation, or recurrent stroke during follow-up, compared with patients < 60 years-of-age.

There are a limited number of studies that present the outcomes after PFO closure in patients older than 60 years with cryptogenic stroke and PFO. *Lee et al.*. presented a retrospective multi-center study comparing PFO closure with medical therapy in patients *≥* 60 years with cryptogenic ischemic stroke and PFO (*n* = 437 of which 161 underwent PFO closure, median 3.9 years follow-up), focusing on recurrent stroke, TIA, and atrial fibrillation (13). They concluded that elderly patients with cryptogenic stroke and PFO had a high rate of recurrent ischemic stroke or TIA, and that device closure was associated with a significant risk reduction, especially in those with a high-risk PFO (13). Further, they concluded that the rate of atrial fibrillation was higher in the PFO closure group (HR 2.28) (13). *Ben-Assa et al.*. presented a retrospective single-center study from U.S.A evaluating 184 patients older than 60 years that underwent PFO closure with median follow-up 3.6 years (14). They presented low rates of recurrent neurologic events similar to younger patients, but a higher incidence of developing atrial fibrillation in the older group (7.6% vs. 2.7%; *P* = 0.007). *Wintzer-Wehekind et al.*. compared the long-term safety and efficacy of PFO closure in patients older than 60 (*n* = 90) with those younger than 60 years (*n* = 385) during a mean follow-up-time of 8 years (17). The study showed that PFO closure was safe with a low rate of ischemic events at long-term also in older patients (17). However, a tendency of higher incidence of recurrent stroke and TIA among the older patients was noted, possibly related to a higher burden of cardiovascular risk factors (17). In our study, the *≥* 60 years-of-age group had similar cardiovascular comorbidities, although they had a higher prevalence of deep venous thrombosis, compared with the patients under 60 years-of-age. The higher prevalence of deep venous thrombosis imposes an increased risk of paradoxical emboli and hence potential relevance of PFO closure in this group. This is supported by *Mattle et al.*., suggesting that changes in the endothelial and platelet function, comorbidity, and reduced physical activity contribute to increased risk of deep vein thrombosis and paradoxical emboli favoring PFO closure in the elderly group (11).

In this study, the incidence of recurrent stroke or TIA within one year after the procedure, and paradoxical emboli during follow-up was very low (< 1%) with no difference between the age groups. The results from previous studies comparing the effect of PFO closure in patients with cryptogenic stroke aged < 55 years with those older than 55 years have not been conclusive (18–21). While *Kiblawi et al.* (18). and *Spies et al.* (19). found no difference in recurrent stroke incidence between patients aged > 55 years and *≤* 55 years respectively, *Luermans et al.* (20). and *Scacciatella et al.* (21). showed higher incidences of recurrent stroke in the > 55 years-of-age groups (2.4% versus 0,6% and 4.0% versus 0.3% respectively). Notably, the follow-up durations were shorter in the studies by *Kiblawi et al.* (18). and *Spies et al.* (19), 18 months, while *Luermans et al.* (20). and *Scacciatella et al.* (21). had median 4 and mean 4.5 years follow-up time respectively. The recurrent strokes in the two studies with higher incidence of events and longer follow-up times occurred late, possibly due to new causes in line with the general clinical pictureof the older patient, and not to paradoxical emboli (20, 21). Our study present incidence of stroke or TIA in line, or even lower in the *≥* 60 years of age group, compared with the previously mentioned studies.

Atrial fibrillation is known as the most common adverse event after percutaneous PFO closure (22). The incidence of atrial fibrillation within 3 months after the procedure in this study was low and did not differ between the age groups, in line with several systematic reviews and meta-analyses (22). However, a higher incidence of atrial fibrillation during the whole follow-up period was seen in the *≥* 60 years-of-age group (9.5 per 1,000 person-years, i.e. 0.95 per 100 person-years). The incidence of atrial fibrillation in the general population *≥* 60 years-of-age in Sweden is estimated to 6.9 cases per 1,000 person-years (23). The finding of higher incidence of atrial fibrillation after PFO closure in the *≥* 60 years-of-age group is supported by previous studies of PFO closure including patients > 60 years-of-age that have shown even higher incidences of atrial fibrillation, with incidences of 3.3 and 5.2 per 100 person years, respectively (24, 25). The overall risk for atrial fibrillation is known to increase with age (23). It is also known that PFO closure is associated with a substantially increased risk of procedure-related atrial fibrillation (26). Our results in line with previous studies showing similar results, emphasizes the importance of prolonged electrocardiogram monitoring before referring older patients to PFO closure (24, 25).

The incidence of recurrent stroke was 2.8 per 1,000 person-years in the > 60 years-of-age group, which is comparable with the incidence of ischemic stroke in Swedish adults > 18 years-of-age of 2.3–2.9 per 1,000 person-years (26). The low incidences of new-onset atrial fibrillation and recurrent ischemic stroke in the *≥* 60 years-of-age group, likely reflect the relatively low risk of cardiovascular diseases in this group. The prevalence of cardiovascular comorbidities was low in the study population, also when comparing cardiovascular comorbidities to the general Swedish population. In the general Swedish population, estimated prevalence of atrial fibrillation (3.9%), diabetes (6–7%), heart failure with reduced ejection fraction (2%), ischemic heart disease (3–7%), hyperlipidemia (30–50%), hypertension (25–30%) and renal insufficiency (5–10%) are higher, compared to the corresponding prevalence in the study population, also in the > 60 years-of-age group (27–32). 

No detailed information about the atrial septal anatomy, of PFO size, or increased atrial pressures were available for analysis in our study. Previous studies have described PFO diameter to increase with aging (33), likely reflecting age-related hemodynamic changes, such as elevated right atrial pressure due to pulmonary hypertension or diastolic dysfunction, thereby increasing the risk of higher grade of right-to-left shunt through a larger PFO, hence amplifying embolic risk (34, 35). The increased risk for cryptogenic stroke associated with larger PFO diameter and presence of atrial septal aneurysms has been shown in elderly patients (22, 35, 36). This could possibly contribute to the use of larger devices to treat patients in the older age group in our study. The finding that the GORE^®^ Cardioform Septal Occluder was more frequently used in patients < 60 years of age, whereas the Amplatzer device in patients *≥* 60 years of age likely reflects that some sites during parts of the study period were dedicated users of a certain device, along with temporal and local trends in accepting patients *≥* 60 years of age for PFO-closure. Those circumstances, together with operator dependent factors, are likely to have caused this unbalanced distribution of PFO-devices between age groups, however we have not been able to fully explore this in the present study.

### Strengths and limitations

A strength of this register-based study is the access to data from the Swedish national registry of adult congenital heart disease (SWEDCON). The registry is known for its extensive validation and high coverage of all hospital care related to congenital heart disease in Sweden, constituting a robust basis for our research. Several validation controls have been performed on the registry. The most extensive one from 2016 concluded that the overall concordance between data in SWEDCON and data in medical records was good and the data from the registry was considered reliable for research (37). However, this study confronts the limitations associated with registry-based research. There is a natural risk of incorrect or absent information, coding or classification of diagnoses, interventions, and details associated with patient- or procedural characteristics. Further, some comorbidities that would have been valuable to analyze were not available in the registry, for example there were no data on heart failure with preserved ejection fraction. Echocardiographic examination results were registered in the registry with data obtained from echocardiography examinations in a clinical setting. However, these data had a lower coverage, especially for shunt grade and prevalence of atrial septal aneurysm, why analysis and discussion of such information has been limited. Further, the registry did not provide information about cortical infarction, why the RoPE score (Risk of Paradoxical Embolism), was not able to calculate.

The diagnoses of stroke, and TIA obtained from the Swedish National Board of Health and Welfare’s registry of patient diagnoses, were verified by the centers of procedure, to ensure the diagnosis was correct. However, two centers (in total 6 cases of postprocedural stroke, and 2 cases of post procedural TIA) were not able to report whether the diagnosis was correctly registered. These 8 patients were hence registered as stroke or TIA in this study.

There were no patients registered with postoperative infection related to the procedure. However, a couple of patients had their devices surgically removed due to suspected infection although no infection could be detected in cultures and the patients were therefore not registered with postoperative infection.

One further limitation could be a possible selection bias of the patients older than 60 years. Although PFO closure is not recommended by today’s guidelines, the patients who underwent a PFO closure were selected to undergo this intervention. This might suggest that these patients were considered having a lower risk compared with the general population older than 60 years, although this was not confirmed in the data sets. Notably, the preoperative comorbidities were equally distributed in the age groups.

## Conclusion

This nation-wide study showed low incidence of recurrent stroke, peripheral embolization, and 3-month post-procedural atrial fibrillation after percutaneous PFO closure in patients aged 60 years and older with low preprocedural comorbidity. The patients older than 60 years had no increased incidence of recurrent stroke or TIA compared with patients < 60 years-of-age. However, incidence of atrial fibrillation > 3 months after PFO closure was higher than observed in the general elderly Swedish population. These findings suggest percutaneous PFO closure is a safe and efficient therapy in the reduction of recurrent stroke also in patients older than 60 years with cryptogenic stroke, although pre-procedural atrial fibrillation detection and monitoring should be encouraged in this patient group Fig. 2.

## Electronic Supplementary Material

Below is the link to the electronic supplementary material.

Supplementary Material 1

## References

[1] Hart RG, Diener HC, Coutts SB, Easton JD, Granger CB, O’Donnell MJ, et al. Embolic strokes of undetermined source: the case for a new clinical construct. Lancet Neurol. 2014;13:429–38. 24646875 10.1016/S1474-4422(13)70310-7

[2] Kent DM, Dahabreh IJ, Ruthazer R, Furlan AJ, Reisman M, Carroll JD, et al. Device closure of patent foramen ovale after stroke: pooled analysis of completed randomized trials. J Am Coll Cardiol. 2016;67:907–17. 10.1016/j.jacc. 26916479 10.1016/j.jacc.2015.12.023 PMC4769377

[3] Alsheikh-Ali AA, Thaler DE, Kent DM. Patent foramen ovale in cryptogenic stroke: incidental or pathogenic? Stroke. 2009;40:2349–55. 19443800 10.1161/STROKEAHA.109.547828 PMC2764355

[4] Handke M, Harloff A, Olschewski M, Hetzel A, Geibel A. Patent foramen ovale and cryptogenic stroke in older patients. N Engl J Med. 2007;357:2262–8. 18046029 10.1056/NEJMoa071422

[5] Saver JL, Carroll JD, Thaler DE, Smalling RW, MacDonald LA, Marks DS, et al. Long-term outcomes of patent foramen ovale closure or medical therapy after stroke. N Engl J Med. 2017;377:1022–32. 28902590 10.1056/NEJMoa1610057

[6] Shah R, Nayyar M, Jovin IS, Rashid A, Bondy BR, Fan T-HM, et al. Device closure versus medical therapy alone for patent foramen ovale in patients with cryptogenic stroke: a systematic review and meta-analysis. Ann Intern Med. 2018;168:335–42. 29310136 10.7326/M17-2679

[7] Mas J-L, Derumeaux G, Guillon B, Massardier E, Hosseini H, Mechtouff L, et al. Patent foramen ovale closure or anticoagulation vs. antiplatelets after stroke. N Engl J Med. 2017;377:1011–21. 28902593 10.1056/NEJMoa1705915

[8] Søndergaard L, Kasner SE, Rhodes JF, Andersen G, Iversen HK, Nielsen-Kudsk JE, et al. Patent foramen ovale closure or antiplatelet therapy for cryptogenic stroke. N Engl J Med. 2017;377:1033–42. 28902580 10.1056/NEJMoa1707404

[9] Pristipino C, Sievert H, D’Ascenzo F, Louis Mas J, Meier B, Scacciatella P, et al. European position paper on the management of patients with patent foramen ovale. General approach and left circulation thromboembolism. Eur Heart J. 2019;40:3182–95. Evidence Synthesis Team; EAPCI Scientific Documents and Initiatives Committee; International Experts. Joint Task Force of European Association of Percutaneous Cardiovascular Interventions (EAPCI), European Stroke Organisation (ESO), European Heart Rhythm Association (EHRA), European Association for Cardiovascular Imaging (EACVI), Association for European Paediatric and Congenital Cardiology (AEPC), ESC Working group on GUCH, ESC Working group on Thrombosis, European Haematological Society (EHA), European Underwater and Baromedical Society (EUBS). 30358849 10.1093/eurheartj/ehy649

[10] Li L, Yiin GS, Geraghty OC, Schulz UG, Kuker W, Mehta Z, et al. Incidence, outcome, risk factors, and long-term prognosis of cryptogenic transient ischaemic attack and ischaemic stroke: a population-based study. Lancet Neurol. 2015;14:903–13. 26227434 10.1016/S1474-4422(15)00132-5 PMC5714616

[11] Mattle HP, Saver JL. Patent foramen ovale increases stroke risk in older people. Nat Rev Neurol. 2018;14:573–4. 30054555 10.1038/s41582-018-0050-7

[12] Mazzucco S, Li L, Rothwell PM. Prognosis of cryptogenic stroke with patent foramen ovale at older ages and implications for trials: a population-based study and systematic review. JAMA Neurol. 2020;77(10):1279–87. 32628255 10.1001/jamaneurol.2020.1948 PMC7550974

[13] Lee PH, Kim JS, Song JK, Kwon SU, Kim BJ, Lee JS, et al. Device closure or antithrombotic therapy after cryptogenic stroke in elderly patients with a high-risk patent foramen ovale. J Stroke. 2024;26(2):242–51. 38836271 10.5853/jos.2023.03265 PMC11164578

[14] Ben-Assa E, Kolte D, Sakhuja R, Hung J, Cruz-Gonzalez I, Laish-Farkash A, et al. PFO Closure in Patients Older Than 60 Years: Reconsidering FDA Age-Based Approval Policies. JACC Cardiovasc Interv. 2024;17(19):2317–9. 39415391 10.1016/j.jcin.2024.06.024

[15] Wang AY, Rothwell PM, Nelson J, Saver JL, Kasner SE, Carroll J et al. Patent Foramen Ovale Closure in Older Patients With Stroke: Patient Selection for Trial Feasibility. Neurology. 2024;102(10):e209388. Epub 2024 May 3. Erratum in: Neurology. 2024;103(7):e209727. 10.1212/WNL.0000000000209388 38701403

[16] Caso V, Turc G, Abdul-Rahim AH, Castro P, Hussain S, Lal A, et al. European Stroke Organisation (ESO) guidelines on the diagnosis and management of patent foramen ovale (PFO) after stroke. Eur Stroke J. 2024;9(4):800–34. 38752755 10.1177/23969873241247978 PMC11569559

[17] Wintzer-Wehekind J, Alperi A, Houde C, Côté JM, Del Val D, Côté M, et al. Transcatheter closure of patent foramen ovale in patients older than 60 years of age with cryptogenic embolism. Revista Española de Cardiología (English Edition). 2020;73(3):219–24. 10.1016/j.rec.2019.07.003 31585849

[18] Kiblawi FM, Sommer RJ, Levchuck SG. Transcatheter closure of patent foramen ovale in older adults. Catheter Cardiovasc Interv. 2006;68:136–44. 16755591 10.1002/ccd.20722

[19] Spies C, Khandelwal A, Timmemanns I, Kavinsky CJ, Schrader R, Hijazi ZM. Recurrent events following patent foramen ovale closure in patients above 55 years of age with presumed paradoxical embolism. Catheter Cardiovasc Interv. 2008;72:966–70. 18942060 10.1002/ccd.21737

[20] Luermans JG, Budts W, Ten Berg JM, Plokker HW, Suttorp MJ, Post MC. Comparison of outcome after patent foramen ovale closure in older versus younger patients. EuroIntervention. 2011;7:209–15. 21646063 10.4244/EIJV7I2A35

[21] Scacciatella P, Meynet I, Presbitero P, Giorgi M, Lucarelli C, Zavalloni Parenti D, et al. Recurrent cerebral ischemia after patent foramen ovale percutaneous closure in older patients: a two-center registry study. Catheter Cardiovasc Interv. 2016;87:508–14. 26106024 10.1002/ccd.26053

[22] Apostolos A, Tsiachris D, Drakopoulou M, Trantalis G, Oikonomou G, Ktenopoulos N, et al. Atrial Fibrillation After Patent Foramen Ovale Closure : Incidence, Pathophysiology, and Management. J Am Heart Assoc. 2024;19:e034249. 10.1161/JAHA.124.034249 PMC11179870 38639354

[23] Lindberg T, Wimo A, Elmståhl S, Qiu C, Bohman DM, Sanmartin Berglund J. Prevalence and Incidence of Atrial Fibrillation and Other Arrhythmias in the General Older Population: Findings From the Swedish National Study on Aging and Care. Gerontol Geriatr Med. 2019;5:2333721419859687. 31276022 10.1177/2333721419859687 PMC6598326

[24] Farjat-Pasos JI, Guedeney P, Horlick E, Abtan J, Nombela-Franco L, Hibbert B, et al. Determinants of adverse outcomes following patent foramen ovale closure in elderly patients. EuroIntervention. 2024;20(16):1029–38. 39155753 10.4244/EIJ-D-24-00156 PMC11317834

[25] Himelfarb JD, Shulman H, Olesovsky CJ, Rumman RK, Oliva L, Friedland J, et al. Atrial fibrillation following transcatheter atrial septal defect closure: a systematic review and meta-analysis. Heart. 2022;108(15):1216–24. 34675040 10.1136/heartjnl-2021-319794

[26] Skibsted CV, Korsholm K, Pedersen L, Bonnesen K, Nielsen-Kudsk JE, Schmidt M. Long‐term risk of atrial fibrillation or flutter after transcatheter patent foramen ovale closure: a nationwide Danish study. Eur Heart J. 2023;44:3469–77. 37279491 10.1093/eurheartj/ehad305

[27] Carlsson M, Asplund K, Norrving B, Stegmayr B, Riksstroke Study Group. Epidemiology of first and recurrent ischemic stroke in Sweden 2010–2019: a Riksstroke study. Neuroepidemiology. 2024;56(6):433–43. 10.1159/000527373 PMC9945185 36223758

[28] Region Östergötland. Diabetes vårdprogram [Diabetes care program]. Medicinska specialistkliniken. 2025. Guideline document no. 11947. Approved by Carl-Johan Östgren. https://ledsys.lio.se/Document/Document?DocumentNumber=11947 Accessed 6 Jan 2026.

[29] Musella F, Rosano GMC, Hage C, Benson L, Guidetti F, Moura B, et al. Patient profiles in heart failure with reduced ejection fraction: prevalence, characteristics, treatments and outcomes in a real-world heart failure population. Eur J Heart Fail. 2023;25(8):1246–53. 37210605 10.1002/ejhf.2892

[30] Wang R, Fratiglioni L, Liang Y, Welmer AK, Xu W, Mangialasche F, et al. Prevalence, pharmacological treatment, and control of cardiometabolic risk factors among older people in central Stockholm: a population-based study. PLoS One. 2015;10(3):e0119582. 25799502 10.1371/journal.pone.0119582 PMC4370718

[31] Swedish Council on Health Technology Assessment. Moderately Elevated Blood Pressure: A Systematic Review [Internet]. Stockholm: Swedish Council on Health Technology Assessment (SBU); 2008 Sep. SBU Yellow Report No. 170/1U.). 28876740

[32] Tomasoni D, Vitale C, Guidetti F, Benson L, Braunschweig F, Dahlström U, et al. The role of multimorbidity in patients with heart failure across the left ventricular ejection fraction spectrum: data from the Swedish Heart Failure Registry. Eur J Heart Fail. 2024;26(4):854–68. 38131248 10.1002/ejhf.3112

[33] Mazzucco S, Li L, Rothwell PM. Prognosis of cryptogenic stroke with patent foramen ovale at older ages and implications for trials: a population-based study and systematic review. JAMA Neurol. 2020;77(10):1279–87. 32628255 10.1001/jamaneurol.2020.1948 PMC7550974

[34] Xu L, Zhou C, Pan X, Zhou J, Sun H, Xu T. Effect of ASA on the risk of cerebrovascular ischemic events in patients with PFO. Ann Clin Transl Neurol. 2022;9(9):1384–91. 35894517 10.1002/acn3.51638 PMC9463951

[35] Wang R, Qiao M, Song G, Wang W, Yang L, Lin Z, Meng L. Predictive value of patent foramen ovale diameter for cryptogenic stroke and age-related differences. Front Cardiovasc Med. 2025;12:1647313. 40918181 10.3389/fcvm.2025.1647313 PMC12408557

[36] Hagen PT, Scholz DG, Edwards WD. Incidence and size of patent foramen ovale during the first 10 decades of life: an autopsy study of 965 normal hearts. Mayo Clin Proc. 1984;59(1):17–20. 6694427 10.1016/s0025-6196(12)60336-x

[37] Bodell A, Björkhem G, Thilén U, Naumburg E. National quality register of congenital heart diseases – can we trust the data? J Congenit Heart Dis. 2017;1:11.

