# Supplementary material for: Percutaneous closure of patent foramen ovale after cryptogenic stroke – a comparison between patients < 60 versus ≥ 60 years-of-age
Source: Cardiovasc Interv Ther. 2026 Apr 29;41(3):816–25. doi: 10.1007/s12928-026-01269-z (PMC13279284; doi:10.1007/s12928-026-01269-z)
Supplement: Supplementary file 1 — Supplementary Material 1 [file 12928_2026_1269_MOESM1_ESM.docx]

**Supplementary information**

**Table S1. ICD codes used for preexisting atrial fibrillation and post-procedural incidence of recurrent cerebral infarction, TIA, peripheral embolization, and atrial fibrillation.**

| \| I48.0 Paroxysmal atrial fibrillation \| \| --- \| \| I48.1 Persistent atrial fibrillation \| \| I48.2 Chronic atrial fibrillation \| \| I48.3 Typical atrial flutter \| \| I48.4 Atypical atrial flutter \| \| I48.9 Atrial fibrillation and atrial flutter, unspecified \| \| G45.0 Vertebro-basilar artery syndrome \| \| G45.1 Carotid artery syndrome (hemispheric) \| \| G45.2 Multiple and bilateral precerebral artery syndromes \| \| G45.3 Amaurosis fugax \| \| G45.4 Transient global amnesia \| \| G45.8 Other transient cerebral ischemic attacks and related syndromes \| \| G45.9 Transient cerebral ischemic attack, unspecified \| \| I74.0 Embolism and thrombosis of abdominal aorta \| \| I74.1 Embolism and thrombosis of thoracic aorta \| \| I74.2 Embolism and thrombosis of arteries of upper extremities \| \| I74.3 Embolism and thrombosis of arteries of lower extremities \| \| I74.4 Embolism and thrombosis of arteries of extremities, unspecified \| \| I74.5 Embolism and thrombosis of iliac artery \| \| I74.8 Embolism and thrombosis of other arteries \| \| I74.9 Embolism and thrombosis of unspecified artery \| \| I63.0 Cerebral infarction due to thrombosis of precerebral arteries \| \| I63.1 Cerebral infarction due to embolism of precerebral arteries \| \| I63.2 Cerebral infarction due to unspecified occlusion or stenosis of precerebral arteries \| \| I63.3 Cerebral infarction due to thrombosis of cerebral arteries \| \| I63.4 Cerebral infarction due to embolism of cerebral arteries \| \| I63.5 Cerebral infarction due to unspecified occlusion or stenosis of cerebral arteries \| \| I63.6 Cerebral infarction due to cerebral venous thrombosis, nonpyogenic \| \| I63.8 Other cerebral infarction \| \| I63.9 Cerebral infarction, unspecified \| |
| --- | --- | --- | --- | --- | --- | --- | --- | --- | --- | --- | --- | --- | --- | --- | --- | --- | --- | --- | --- | --- | --- | --- | --- | --- | --- | --- | --- | --- | --- | --- |

**Table S2. Distribution of interatrial shunts in patients with cryptogenic stroke undergoing percutaneous PFO closure**

|  | **All patients, n=733** | **>60 years-of-age,**  **n=91** | **<60 years-of-age, n=642** | **p-values** |
| --- | --- | --- | --- | --- |
| Left-to-right shunt, n (%)  Small, n  Moderate, n  Large, n | 187 (25.5)  164  20  3 | 28 (30.8)  26  1  1 | 159 (24.8)  138  19  2 | 0.219 |
| Right-to-left shunt, n (%)  Small, n  Moderate, n  Large, n | 546 (74.5)  236  205  105 | 63 (69.2)  29  25  9 | 483 (75.2)  207  180  96 | 0.219 |

In total 733 (66.6%) patients were registered with an echocardiography evaluation of the shunt grade in the national registry.

**Table S3. Distribution of device sizes**

| **Device size (mm)** | **All patients, n=1,101** | **>60 years-of-age,**  **n=134** | **<60 years-of-age, n=967** |
| --- | --- | --- | --- |
| **18, n (%)** | 52 (4.7) | 12 (9.0) | 40 (4.1) |
| **20, n (%)** | 56 (5.1) | 5 (3.7) | 51 (5.3) |
| **25, n (%)** | 593 (53.9) | 59 (44.0) | 534 (55.2) |
| **30, n (%)** | 173 (15.7) | 20 (14.9) | 153 (15.8) |
| **35, n (%)** | 127 (11.5) | 27 (20.1) | 100 (10.3) |
| **Unknown size, n (%)** | 76 (6.9) | 10 (7.5) | 66 (6.8) |
